# Etiologies and Outcomes Following Duodenal Perforation in Acute Peritonitis: A Systematic Review

**DOI:** 10.7759/cureus.74707

**Published:** 2024-11-28

**Authors:** Rajeev Shahi, Nurul Haque Siddiqui, Imran Ahmed Khan, MD. Abu Bashar

**Affiliations:** 1 Surgery, Autonomous State Medical College, Kushinagar, IND; 2 Anesthesiology, Balrampur Hospital, Lucknow, IND; 3 Community Medicine, Baba Raghav Das Medical College, Gorakhpur, IND; 4 Community and Family Medicine, All India Institute of Medical Sciences, Gorakhpur, IND

**Keywords:** acute peritonitis, duodenal perforation, outcomes, peptic ulcer disease, surgical intervention, trauma

## Abstract

Duodenal perforation often presents as an acute onset of abdominal pain and potential complications such as systemic infection, multiple organ system failure, and even death. It can result from various causes, including peptic ulcer disease (PUD), trauma, malignancies, and infections. Prompt diagnosis and timely intervention are critical for better outcomes, though mortality can be high, particularly in delayed cases. This systematic review aims to synthesize available literature on the etiologies and outcomes associated with duodenal perforation presenting as acute peritonitis, offering a comprehensive overview for guiding effective management strategies.

A systematic search was conducted across electronic databases including PubMed, CINAHL, and Google Scholar to identify relevant studies published up to August 2024. Inclusion criteria comprised observational studies, case reports, and case series on duodenal perforation in acute peritonitis. Review articles and non-English language studies were excluded. Two reviewers independently performed data extraction with the opinion of a third reviewer to resolve controversies. Information was gathered on study characteristics, patient demographics, etiology, treatment, and outcomes.

A total of 18 studies with 536 participants were included, encompassing a diverse patient population. The primary etiologies identified were PUD, trauma, foreign body, and iatrogenic causes. Treatment approaches ranged from conservative management to surgical interventions, with outcomes varying based on the underlying cause and timeliness of treatment. Postoperative complications were significant, including wound infections, anastomotic leaks, and, in severe cases, multiorgan failure. Mortality was largely associated with delayed intervention. Despite advancements in surgical techniques, the condition still carries a significant risk of complications and mortality, underscoring the need for timely and effective medical care. Future research should focus on developing standardized guidelines to optimize the management of duodenal perforations and reduce associated morbidity and mortality.

## Introduction and background

Duodenal perforation most often manifests as acute peritonitis and is characterized by sudden onset of abdominal pain and other clinical features. It may accompany gastrointestinal bleeding with systemic signs of infection and can lead to severe complications if go unrecognized and not managed appropriately [1]. The duodenum is the first segment of the small intestine that continues with the stomach. It is prone to perforation from various etiologies, peptic ulcer disease (PUD) being the most common cause among them [2]. Other significant causes include trauma, malignancies, infections particularly typhoid and tuberculosis, ischemia, diverticula, and some autoimmune diseases [3-5]. Duodenal perforation occurs more commonly among males, with individuals aged between 19 and 45 years and from lower socioeconomic status [6].

Free intraperitoneal gas on abdominal plain film in an erect position is a direct sign of perforation, while intraperitoneal free fluid and reduced intestinal peristalsis are indirect signs. Abdominal ultrasound or computed tomography (CT) scans efficiently diagnose duodenal perforation [7]. Diagnosis can be delayed in atypical cases and when symptoms are masked by other conditions, such as preeclampsia in pregnant women [8]. Prompt recognition and effective management are crucial to prevent severe complications and unfavorable outcomes.

Wound infection, anastomotic leak, burst abdomen, pneumonia, septicemia, and acute renal failure are among some common postoperative complications. In rare cases, patients may present with an anterior abdominal abscess or altered neurological status [9,10]. Mortality rates vary but can be as high as 10%. Septic shock and multiorgan failure are the leading causes of death [9]. Understanding the causes and outcomes of this condition is crucial for effective management and improving patient outcomes. This systematic review aims to comprehensively analyze the etiologies and outcomes of duodenal perforation presenting as acute peritonitis.

## Review

Search strategy

A systematic search of the literature was conducted in PubMed, CINAHL, and Google Scholar to identify studies published since inception up to August 2024. The search terms included "duodenal perforation", "acute peritonitis", "etiologies", and "outcomes" in various combinations using Boolean operators. The search was limited to human studies published in the English language.

Inclusion and exclusion criteria

Studies reporting on patients with duodenal perforation presenting as acute peritonitis, studies detailing etiology and outcome with or without treatment methods, observational studies, case reports, and case series were included in this review.

Editorials, commentary, perspectives, opinion papers, review articles, studies focusing on other types of intestinal perforations, non-English language publications, and animal studies were excluded. 

Data extraction and analysis

Data were extracted independently by two reviewers IAK and NH using a standardized form. Extracted information included study characteristics, patient demographics, etiology, treatment modalities, and outcomes. Discrepancies were resolved through discussion among the reviewers and the third reviewer MAB.

Results

**Figure 1:**
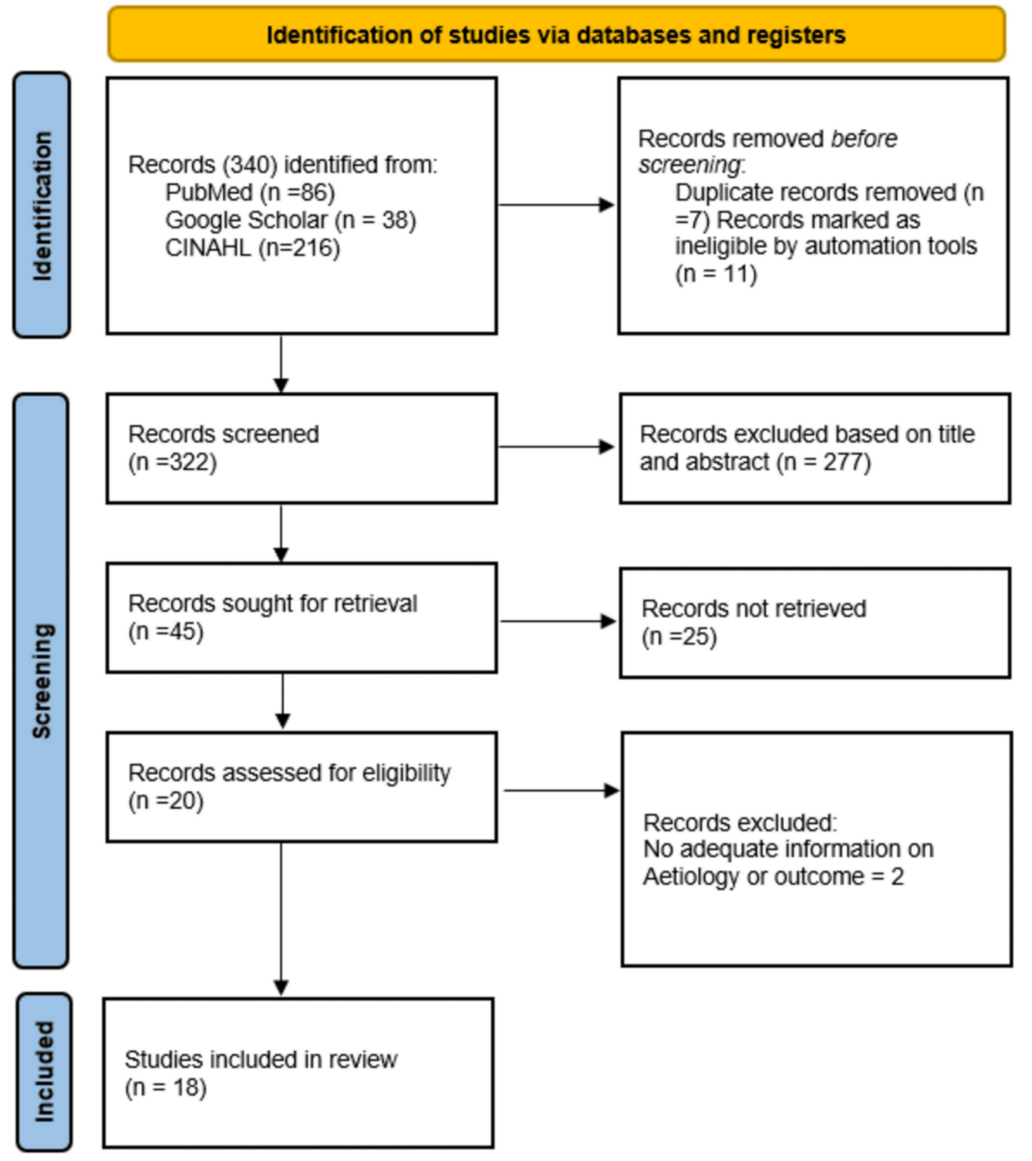
PRISMA flow diagram of articles selected. PRISMA, Preferred Reporting Items for Systematic Reviews and Meta-Analyses

A total of 340 potentially relevant articles were identified from PubMed (86), CINAHL (216), and Google Scholar (38) of which 322 were available for title and abstract screening. The screening of titles and abstracts rejected 277 studies and 45 articles were retrieved for full-text after the reviewers’ approval based on the selection criteria, of which full texts of 25 articles could not be retrieved and a further two articles were excluded due to inadequate information at this stage. Finally, 18 articles were selected for this systematic review comprising a total of 536 participants. The Preferred Reporting Items for Systematic Reviews and Meta-Analyses (PRISMA) 2020 flow diagram depicting the search, screening, and inclusion is presented in Figure 1.

Selected articles and the summary of the results are presented in Table 1. The included studies span a wide range of geographical locations, including Canada, the USA, the UK, Spain, India, Italy, Japan, Saudi Arabia, Taiwan, France, Turkey, China, and Pakistan. The studies comprised case reports, retrospective studies, and case series, with sample sizes ranging from a single case to 344 patients.

**Table 1:** Summary of the included articles. PD, peritoneal dialysis; VAC, vacuum-assisted closure; FBs, foreign bodies

Sr No.	Author (year)	Country	Article type	Sample size	Summary
1	Booth and Williams (1971) [11]	Canada	Case report	344	A 10-year experience of 344 patients undergoing surgical treatment of perforated duodenal ulcer with a mortality of 2.9% was reported. The major causes of death were peritonitis, cardiopulmonary complications, and gastrointestinal bleeding.
2	Berne and Donovan (1989) [12]	United States	Retrospective study	35	Perforation of a duodenal or pre-pyloric ulcer was treated nonoperatively in 35 adult patients. They presented with pneumoperitoneum with clinical evidence of peritonitis. The mortality rate was found 3%.
3	Eden and Williams (1992) [13]	United Kingdom	Case report	1	Duodenal perforation complicating laparoscopic cholecystectomy was reported. Prolonged shoulder tip pain should be investigated for the possibility of subphrenic collection.
4	Flores et al. (2006) [14]	Spain	Case report	1	A two-month-old infant weighing 3.5 kg, with alveolar interstitial pneumopathy on mechanical ventilation,developed a duodenal perforation due to a 6-Fr polyurethane transpyloric tube. The perforation was sutured, but the patient developed new intestinal perforation. Endoscopic or contrast radiographic evaluation must be performed in case of doubt.
5	Kalawat and Sharma (2010) [15]	India	Case report	1	Fungal peritonitis is most commonly caused by Candida species. However, prophylactic use of fluconazole in patients prone to recurrent gastrointestinal perforations or anastomotic leaks has significantly reduced the incidence of Candida peritonitis. Consequently, rare opportunistic fungi are becoming increasingly prevalent. Here, we present a case of Trichosporon peritonitis following duodenal perforation.
6	Cascio et al. (2011) [16]	Italy	Case report	1	A 62-year-old man with Candida krusei peritonitis after duodenal perforation was successfully treated with a 14-day course of caspofungin.
7	Shen et al. (2011) [17]	United States	Case report	1	A 55-year-old man presented with a history of three weeks of sharp epigastric pain radiating to the right upper quadrant, fever, and generalized weakness. Investigations showed a proximal duodenal perforation, which was drained, and systemic antibiotics were given. This conservative management resulted in the healing of the ulcer and early closure of the enteric fistula.
8	Nishino et al. (2012) [18]	Japan	Case report	1	A 77-year-old-man on peritoneal dialysis developed lower abdominal pain and cloudy effluent. Escherichia coli was detected in the PD effluent culture. An abdominal computed tomography scan showed a fish bone in the duodenal wall which was removed by upper gastrointestinal endoscopy. A careful investigation of the possibility of enteric peritonitis from the intestinal tract is recommended when E. coli is detected in effluent cultures during PD.
9	Issa et al. (2013) [19]	Saudi Arabia	Case report	1	Endoscopic retrograde cholangiopancreatography may lead to migration of biliary stents, which rarely presents as intestinal perforation. A case of a migrated biliary stent is presented that resulted in duodenal perforation and biliary peritonitis.
10	Chao et al. (2014) [20]	Taiwan	Case report	1	Immunocompromised patients with intravenous catheters may rarely develop Rhizobium radiobacter infection. Peritonitis reported in these patients has been attributed to intra-peritoneal devices undergoing peritoneal dialysis. A case of perforated ulcer complicated by peritonitis caused by R. radiobacter in a healthy adult is reported, which was treated successfully with surgery and antibiotics.
11	Gupta et al. (2015) [21]	Canada	Case report	1	A seven-year-old boy presented in septic shock secondary to appendicitis with generalized peritonitis. Following initial optimization, he underwent a series of surgeries. There was also a perforated duodenal ulcer. A tailored treatment approach resulted in a favorable outcome.
12	Nobori et al. (2016) [22]	Japan	Case report	2	For giant duodenal ulcers with poor general condition, simple closure and reconstruction with omental patching may not be appropriate treatment. Antrectomy with gastric disconnection (gastrostomy, duodenostomy, feeding jejunostomy, and cholecystectomy) is recommended.
13	Prevot et al. (2016) [23]	France	Case report	1	Pleuroperitoneal communication is often asymptomatic but sometimes causes hydrothorax. This report provides insight into an unreported association of concomitant pleural effusion and acute infectious abdominal disease, due to perforated duodenal ulcer. Pleural effusion with acute abdominal pain may signal a pleuroperitoneal communication requiring surgical management.
14	Garside et al. (2018) [24]	United Kingdom	Case report	1	Duodenal perforation with common bile duct rupture secondary to blunt handlebar trauma in an 11-year-old boy was reported. The patient presented with upper abdominal wall ecchymosis, pain, and vomiting. He was discharged after initial treatment after improvement but presented later with signs of peritonitis. Duodenal perforation was seen on computed tomography, which was confirmed during laparotomy where common bile duct rupture was also demonstrated. Primary repair of the duodenum was successfully undertaken.
15	Hadano et al. (2018) [25]	Japan	Case report	1	A 75-year-old Japanese male patient presented in septic shock with duodenal perforation and secondary peritonitis. Blood cultures on admission were positive for Gram-positive and Gram-variable cocci, and Gemella hemolysans was identified. The patient was successfully treated with a two-week course of antibiotics.
16	Eğin et al. (2019) [26]	Turkey	Case report	1	Severe peritonitis developed owing to duodenal re-leak on the second postoperative day in a 55-year-old male perforated duodenal ulcer patient. Relaparotomy was performed and the necrotic omentum was dissociated from the bulbous duodenum. Since primary source control is often difficult when treating duodenal leaks, the two-way VAC system is a convenient solution for localizing the source of the peritonitis and removing toxic peritoneal material.
17	Ma et al. (2023) [27]	China	Retrospective study	34	Endoscopically diagnosed gastrointestinal FBs from four tertiary hospitals in 12,851 patients in China were retrospectively reviewed. Endoscopic removal of duodenal perforating FBs was found safe and effective by experienced endoscopists. Surgical intervention was required in patients with FBs over 10 cm, both sides perforation, multiple perforating FBs, or severe infections.
18	Malik et al. (2023) [28]	Pakistan	Case series	108	A case series of 108 patients with perforated duodenal ulcers concluded that perforation of duodenal ulcers remains a frequent clinical problem predominantly affecting males. Simple closure with indirect Graham’s Omentopexy was effective with excellent results in the majority of the cases despite patients’ late presentation in our center.

Table 2 shows the reported aetiologies and outcomes from the included articles. Duodenal perforation shows varied aetiologies and outcomes-the majority of patients presented with peritonitis with varying pathogens and commensals.

**Table 2:** Etiologies and outcomes of duodenal perforation.

Etiology	Outcome
Acid peptic disease	Full recovery
Migration of endoscopically placed biliary stents	Peritonitis (bacterial, fungal)
Feeding tube	Gastrointestinal bleeding
Foreign body	Duodenal re-leak after repair
Laparoscopic cholecystectomy	Opportunistic infections
After neurosurgery	Cardiopulmonary complications
Blunt abdominal trauma	Death

Etiology

The primary etiology of duodenal perforation identified across the studies was PUD, with various contributing factors including surgical interventions, endoscopic procedures, foreign body (FB) ingestion, trauma, and infections. Several studies highlighted specific risk factors.

PUD is a common cause of duodenal perforation, with mortality rates ranging from 2.9% to 3% in large cohorts [11,12]. Some surgical and endoscopic Interventions like laparoscopic cholecystectomy [13], and endoscopic biliary stent migration [19], were identified as procedural causes of duodenal perforation. Several cases reported perforation due to ingested FBs, such as fish bones [18], and endoscopic clips [27]. Rare pathogens like Candida krusei [16] and Rhizobium radiobacter [20], were implicated in some cases of peritonitis following duodenal perforation.

Outcomes

Timing of surgical intervention, type of surgical intervention, and method of wound closure play a crucial role in patient outcome [19,22]. Multiple organ systems were found affected in a few cases. Mortality was a concern in delayed diagnosis and treatment of duodenal perforation patients. The outcomes varied widely depending on the underlying etiology, promptness of diagnosis, and the management approach. The mortality rates reported were relatively low, with a significant cohort study showing a 2.9% mortality rate [11], and another reporting 3% [12]. However, mortality was not uniformly reported across all studies. Many studies highlighted the importance of timely surgical intervention. Techniques such as primary repair, omental patching, and in more severe cases, complex procedures like gastric disconnection [22], were utilized. Some studies reported successful outcomes with conservative management, such as sealing perforations and using systemic antibiotics [17]. Cases involving rare pathogens were successfully managed with targeted antimicrobial therapy, which was crucial for improving survival rates [16,25]. Postoperative complications such as leakage of the duodenal ulcer [26], and biliary peritonitis [19], were noted, requiring repeat surgical interventions.

Discussion

The findings of this systematic review highlight the multifactorial etiology of duodenal perforations presenting as acute peritonitis, with PUD remaining the predominant cause highlighting the role of effective management of PUD to prevent perforation. Trauma and malignancy also contribute significantly necessitating a high index of suspicion in appropriate clinical contexts. The variability in outcomes is closely linked to the timeliness of diagnosis, the underlying cause, and the management approach. Early diagnosis, facilitated by prompt imaging, and timely surgical intervention are paramount in improving patient outcomes. Conservative management may be suitable for select cases with contained perforations and stable clinical presentation.

PUD was a leading cause, particularly in older studies, highlighting the role of medical advancements in reducing the incidence of such perforations over time [29,30]. Helicobacter pylori infection and the use of non-steroidal anti-inflammatory drugs (NSAIDs) are leading causes of PUD and subsequent duodenal perforation [31]. Other contributing factors include smoking, psychological stress, and certain medical conditions like Zollinger-Ellison syndrome [32].

The role of surgical intervention remains central to the management of duodenal perforations, with various techniques proving effective in different clinical scenarios. The management approach may include a conservative approach, minimally invasive surgeries, or an urgent exploratory laparotomy tailored to the clinical course of the patient [33-35].

Our analysis found that the use of non-operative management in selected cases suggests that conservative approaches can be considered in well-defined situations, particularly where the perforation is sealed and the patient is stable [12,17]. Endoscopic removal of duodenal perforating FBs was found safe and effective and can be the first choice of treatment for experienced endoscopists [27]. Various surgical techniques are used to treat large duodenal perforations, ranging from simple damage-control operations to complex resections. The choice of technique should be guided by factors such as the location of the perforation, degree of tissue loss, and patient stability [26,28,36]. The mortality rate for duodenal perforation varies widely in the literature depending on the complexity of the cases, patient population, and management strategies with reported rates ranging from 2.9% to 26% [37-39].

The review also brought attention to less common causes of duodenal perforation, such as FB ingestion and rare infections, which require a high index of suspicion and prompt management [18,27]. The successful treatment of cases with rare pathogens like Candida krusei and Rhizobium radiobacter highlights the importance of targeted antimicrobial therapy [16,20].

The review also identifies gaps in the literature, including the need for standardized treatment protocols and larger, multicentre studies to better define optimal management strategies. The heterogeneity of the studies and the variability in treatment approaches highlight the necessity for individualized patient care based on clinical presentation and underlying etiology.

The study has a few limitations requiring a careful interpretation. Potential publication bias, heterogeneity, and the quality of the included studies are a few noticeable limitations of this review.

## Conclusions

Duodenal perforation presenting as acute peritonitis is a medical emergency requiring prompt diagnosis and evidence-based management. PUD remains the leading cause of duodenal perforation followed by trauma, FB, and iatrogenic causes. Early surgical intervention significantly improves outcomes, although conservative management may be appropriate for certain cases. Despite advancements in surgical techniques, the condition still carries a significant risk of complications and mortality, highlighting the need for timely and effective medical care. This systematic review emphasizes the importance of a tailored approach to the management of duodenal perforations, taking into account the underlying etiology, patient condition, and available resources. Future research should focus on establishing standardized treatment protocols and further exploring the role of minimally invasive techniques in the management of duodenal perforations.
